# Launch of the first canine mobile blood donation center in Asia: development, outcomes, and influence of an animal bloodmobile

**DOI:** 10.3389/fvets.2024.1402459

**Published:** 2024-07-12

**Authors:** Hee-Jae Choi, Hyun-Jung Han

**Affiliations:** ^1^Department of Veterinary Emergency and Critical Care, College of Veterinary Medicine, Konkuk University, Seoul, Republic of Korea; ^2^KU Center for Animal Blood Medical Science, Konkuk University, Seoul, Republic of Korea

**Keywords:** canine blood donation, mobile blood drive, bloodmobile, blood donation promotion, canine blood donor, participant satisfaction

## Abstract

**Introduction:**

A mobile blood donation station allows a maximum number of donors to donate blood at any location. In veterinary medicine, no previous studies have reported the use of bloodmobiles for blood donation in animals. We assessed Asia’s first canine mobile blood donation center, which was trialed using a modified vehicle in South Korea.

**Methods:**

A vehicle was modified into a canine bloodmobile with two sections: the front as a laboratory and the back as a blood collection room with necessary equipment. To recruit companion dogs nationwide, the campaign was advertised on television and promoted via social media. Applications of the dogs meeting the following criteria were accepted: in general good health, between 2-8 years old, body weight above 25kg, vaccinated, regularly on heartworm and ectoparasite prophylactics. Pre-donation procedures included medical screening and informed consent, followed by blood collection in a routine fashion. Post-donation, dogs were monitored for complications and owners completed a post-donation survey.

**Results:**

Of the 750 applicants, 48 donor dogs were selected for investigation. Ten failed to donate blood owing to the following issues: behavioral problems (2/48), positive results on vector-borne disease screening tests (5/48), in-tubing clot formation (2/48), and absence on the relevant appointment date (1/48). Blood collection took approximately 12 minutes, and the entire procedure lasted an average of 1.5 hours per donor. The prevalence rates of dog erythrocyte antigen 1-negative and 1-positive blood were 32.6% and 67.4%, respectively. There were no donation-related complications, except for one dog that had contact dermatitis induced by clipper irritation. The post-donation survey completed by 46 owners revealed that most were satisfied with the campaign. The convenience of the mobile blood drive (93.5%) was a key factor contributing to high owner satisfaction and willingness to participate in future campaigns (95.7%), in line with findings from prior veterinary and human blood donation motivation research.

**Discussion:**

The bloodmobile effectively increased engagement in canine blood donation by enhancing accessibility. To optimize canine mobile blood drives, procuring larger vehicles and enhancing infrastructure for future campaigns would be beneficial. In conclusion, this study showed that Asia’s first canine bloodmobile was successful in terms of improving the convenience, accessibility, and efficacy of canine blood donation. Although the concept is still unfamiliar to the public, active promotion of canine blood donation can help ensure a robust blood donation culture in the veterinary field.

## 1 Introduction

A bloodmobile is a mobile unit stocked with the equipment and supplies necessary for blood donation procedures, which travels from place to place with medical staff who collect blood (1). The bloodmobile was first introduced for the service of humanity in February 1941 by Dr. Charles Drew, the medical director of the American Red Cross’s initial collection program, to meet the increased demand for blood products and promote a culture of blood donation (2). The greatest advantage of bloodmobiles is that they provide potential donors with easier access to blood donation centers by minimizing their travel time, thereby motivating the participation of local residents. With a large truck, bus, or van equipped with beds, tables, refrigerators, and supplies for blood collection, the blood bank could be brought to donors, saving them the trouble of traveling. Currently, bloodmobiles function as the most modern and effective facility for blood donation and are considered an asset for the augmentation of voluntary blood donation. They also ensure stable collection for better provision of blood and blood components (3).

In dogs, the number of critical patients in need of blood transfusion is steadily increasing, as is the demand for blood products, leading to the demand for an increasing number of canine blood donors to establish a steady blood supply (4, 5). While the proportion of canine donors is unknown, it is likely to be very small, and the demand for blood products could be greater than the available supply; furthermore, veterinary volunteer donor programs continuously report donor shortages (6, 7). In South Korea, blood supply is highly dependent on private organizations and the dogs owned by them rather than on pets. Concerns about animal bioethics, unsanitary environments, limited blood supply, and the quality of blood products have arisen. As a result, there needs to be greater engagement and reassurance regarding animal bioethics, controlling blood shortages, encouraging blood donation, and inducing an increasing number of canine donors to participate. To increase the number of suitable canine donors and their recruitment, we aimed to understand how donation could be made more convenient, as this is the most common motivation for human blood donors (8–10). Based on blood donation trends in humans, bloodmobiles could be expected to enhance donor participation, saving time and reducing the burden on potential donors and their owners.

Against this background, we aimed to investigate the outcomes and efficacy of a bloodmobile designed and operated for canine blood donation. We hypothesized that a well-equipped bloodmobile with well-trained medical staff would enable a more convenient blood donation experience and increase the comfort of canine donors and satisfaction of their owners regarding blood donation. To the best of our knowledge, this is the first assessment of a canine bloodmobile in not only South Korea but also all of Asia.

## 2 Materials and methods

### 2.1 Vehicle modification

**Figure 1:**
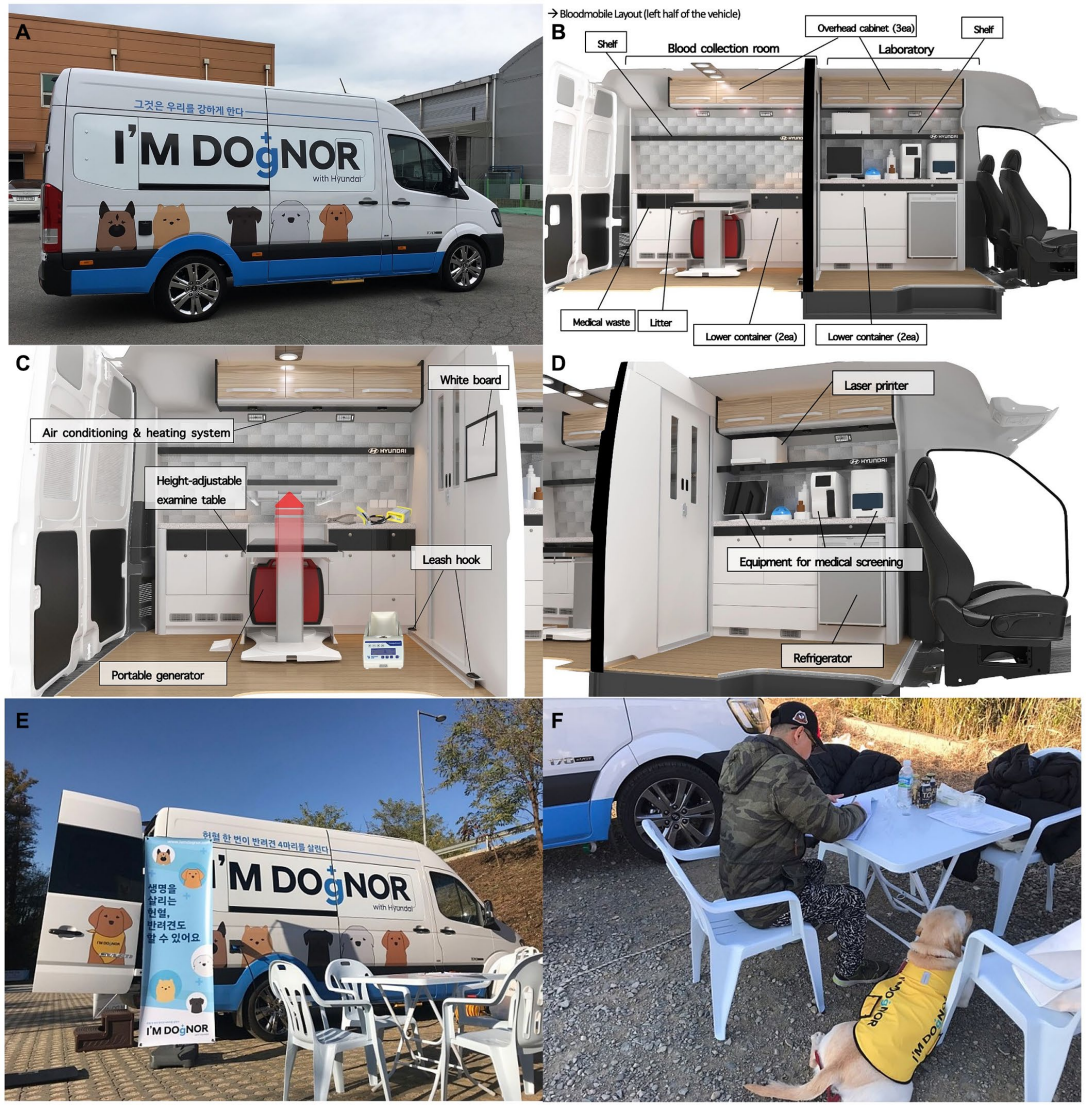
Exterior and interior of the bloodmobile. **(A)** The exterior design of the bloodmobile (Solati, Hyundai Motors Co., Seoul, Korea). The external appearance of the blood donation vehicle features a campaign slogan that refers to canine blood donors, with the phrase “I’M DOgNOR” prominently displayed on the window level. Additionally, an illustration highlights a variety of large dog breeds on the lower portion of the vehicle. **(B)** The interior design layout of the bloodmobile. The front and back sections were used as the laboratory and the blood collection room, respectively. **(C)** The rear compartment of the bloodmobile. This was used as both a pre-donation examination room and a blood collection room. This compartment was equipped with a height-adjustable examination table and devices for blood collection such as a scale, tube sealer, tube stripper, blood collection bags, and medical supplies. **(D)** The front compartment of the bloodmobile. This functioned as a medical laboratory and was equipped with medical screening devices such as hematology, serum chemistry, and coagulation analyzers; a refrigerator, and supplies required for pre-blood collection examinations. **(E,F)** A temporary consulting room set up outside the vehicle.

A vehicle, namely a Solati (Hyundai Motor Company Inc., Seoul, South Korea), was selected and modified to include two compartments (Figures 1A–D). Two vehicle modification specialists, two employees from the Hyundai Motor Company, and two veterinarians from emergency and critical care departments collaborated to design and renovate the vehicle.

The front compartment served as a medical laboratory for pre-donation blood tests, while the rear compartment functioned as both a pre-donation examination area and a blood collection room. Owing to the limited space inside the vehicle, a temporary consulting room was set up outside (Figures 1E,F). Effective promotion was additionally achieved at the scene through the visibility of the bloodmobile to pedestrians and acquaintances of the donors.

### 2.2 Donor recruitment and selection

**Figure 2:**
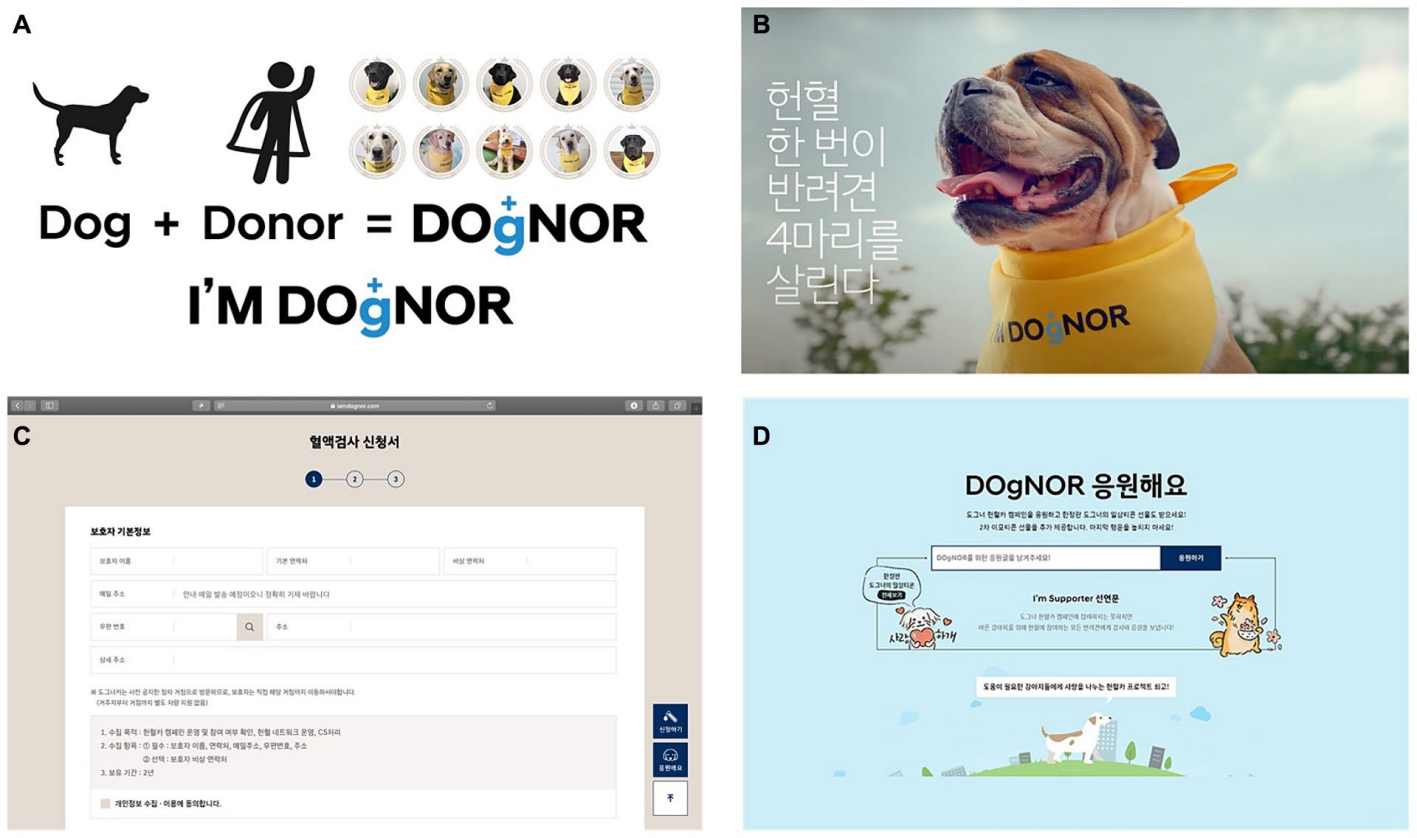
Various means were employed to promote the canine mobile blood drive. **(A)** A campaign slogan focused on canine blood donors; **(B)** A series of television advertisements were created, broadcast, and uploaded to various social media platforms such as YouTube and Instagram; **(C)** A website page for blood donation applicants; **(D)** A website page for supporting comments.

Dog owners’ awareness of canine blood donation in South Korea had not been assessed before the present campaign. To recruit donors from diverse regions and introduce the concept of blood donation by companion dogs to the public, the campaign was advertised using various means. Multiple modes of communication, such as television commercials, websites, and social media, were used for campaign promotion. As the concept of blood donation by companion dogs is unfamiliar in South Korea, various promotional approaches had to be developed to recruit donors nationwide. A new term for “canine donor”—“DOgNOR”—which combines the words “dog” and “donor”—was created, and the campaign was titled “I’M DOgNOR.” For promotion and donor recruitment, a website was created, and advertisements were produced and provided by the Hyundai Motor Company, featuring experienced donor dogs. The advertisements were broadcast on television and posted on social media platforms, including YouTube and Instagram (Figure 2).

Regarding the typical considerations in donor selection, applications from the owners of any healthy dog between the ages of 2 and 8 years, weighing more than 25 kg, were accepted if the potential donor was not on any medication, was fully vaccinated, and was regularly administered preventive measures for heartworms, fleas, and ticks (11). Conventionally, the age of canine blood donors ranges from 1 to 8 years (11). However, in this study, the lower age limit was revised from 1 year to 2 years to ensure that the donors are fully grown and past the vulnerable stages of their adolescent development, thereby reducing the risks associated with blood donation, such as hypotension (11, 12). As potential applicants were distributed throughout the country, pre-donation medical screening was performed for all participants on-site on the day of the campaign.

### 2.3 Campaign schedule and location

**Figure 3:**
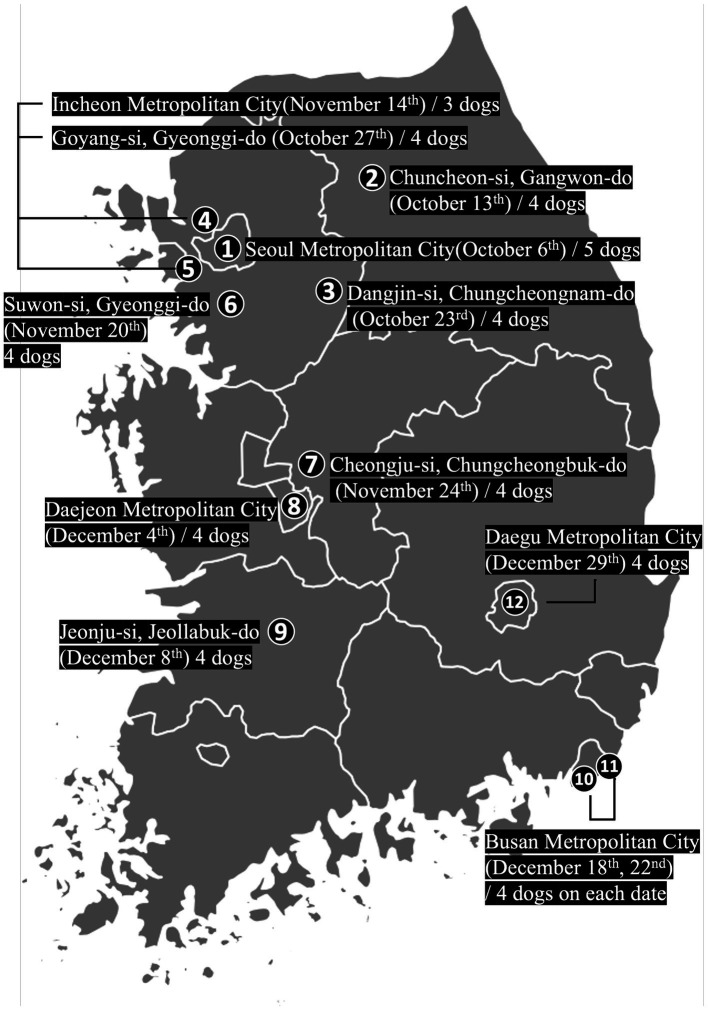
Weekly locations of the bloodmobile in the blood donation campaign held in the Republic of Korea from October to December 2019. The marked areas show the 12 regions visited by the bloodmobile and the number of donor dogs in that region.

The bloodmobile visited 12 locations distributed throughout the country according to the campaign schedule. The campaign spanned a total of 12 weeks from October to December 2019 (Figure 3).

### 2.4 Blood donation procedure

#### 2.4.1 Pre-donation procedure

A temporary consulting room was installed adjacent to the bloodmobile. The veterinarian described the blood donation process in detail to the participating owners, explained the need for medical screening, warned of possible complications, and took a brief history of the donor. Informed consent was obtained from all owners (Figures 1E,F). Afterward, the donor entered the bloodmobile through the doors at the back.

After the donor entered the blood collection room, a thorough physical examination was performed, hair was clipped from the venipuncture site, and a topical anesthetic cream was applied.

The following tests were performed as part of the medical screening to check for vector-borne pathogens: complete blood count, serum chemistry (albumin, albumin/globulin, alkaline phosphatase, alanine aminotransferase, blood urea nitrogen, blood urea nitrogen/ creatinine, globulin, glucose, and total protein), blood typing for dog erythrocyte antigen (DEA) 1 (Quick Test BT Canine, Alvedia, Lyon, France), prothrombin, activated partial thromboplastin time, and SNAP^®^ 4Dx^®^ Plus. The vector-borne pathogens tested for included heartworm, Lyme disease (*Borrelia burgdorferi* infection), Ehrlichia (*Ehrlichia canis, Ehrlichia ewingii*, or *Ehrlichia chaffeensis*), and Anaplasma (*Anaplasma phagocytophilum* and *Anaplasma platys*). In addition to on-site medical screening, IDEXX’s Tick/Vector Comprehensive RealPCR™ Panel–Canine was conducted to detect *Anaplasma* spp., *Babesia* spp., *Bartonella* spp., Canine *Hemotropic Mycoplasma*, *Ehrlichia* spp., *Hepatozoon* spp., *Leishmania* spp., quantitative *Neorickettsia risticii*, and *Rickettsia* spp. Owners were notified of the polymerase chain reaction results over the phone.

While the local anesthetic cream was being absorbed, the donor waited outside for approximately 15 min with their family. The veterinarians interpreted the bloodwork results at the front section of the bloodmobile (laboratory), explaining the results to the owner when the pre-donation examination was completed.

#### 2.4.2 Blood collection and post-donation care

**Figure 4:**
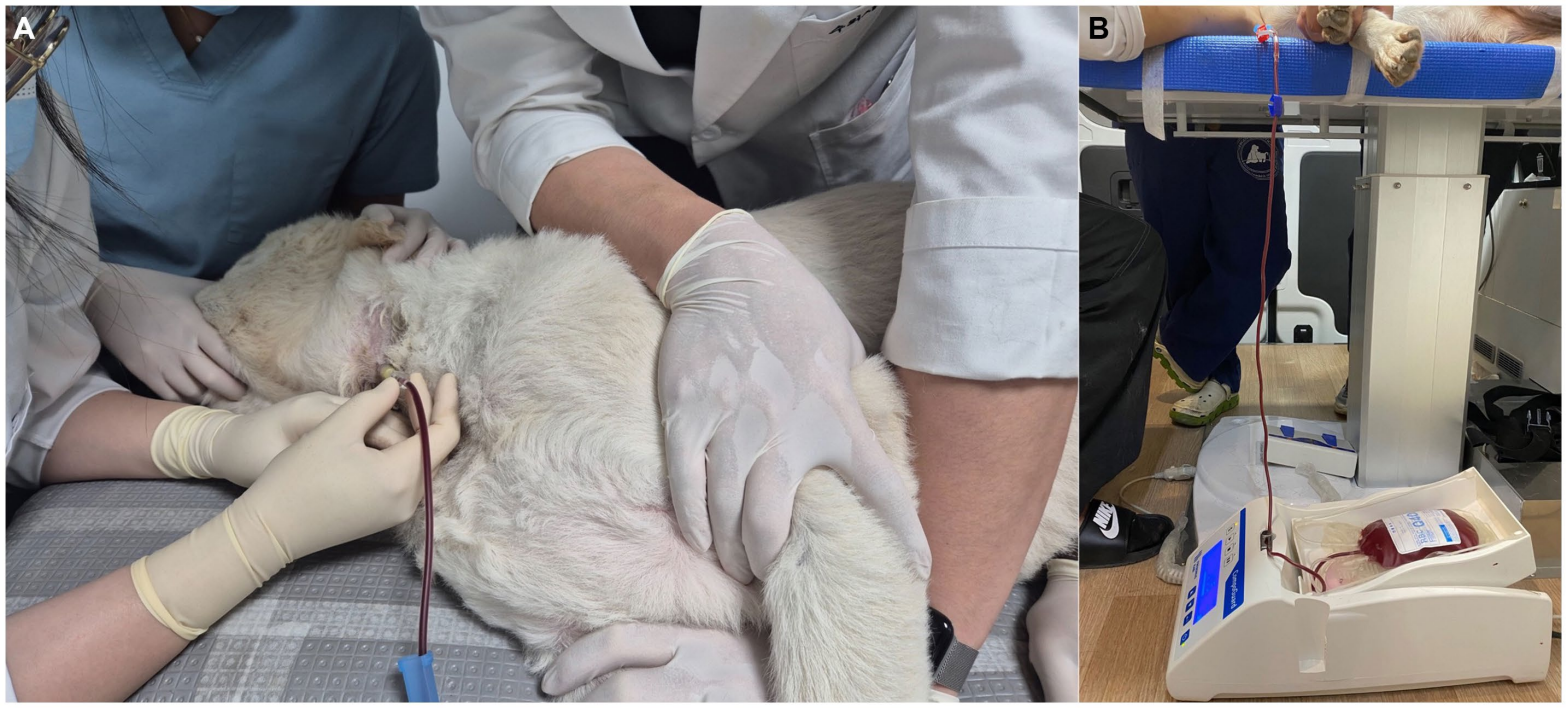
Scenes from the campaign. **(A)** Blood collection performed inside the bloodmobile. **(B)** A blood bag on the blood scale mixer.

Blood was collected in a routine fashion, with the donor in lateral recumbency, using gravity-assisted collection in a closed system. At each blood collection, the veterinary staff ensured a suitable mixing of blood and anticoagulants in the primary blood collection bag. The blood mixing apparatus utilized was CompoGuard^®^ basic (Fresenius Kabi AG). The blood mixing equipment was designed to effectively mix blood with anticoagulants to prevent excessive bleeding or low-volume blood bags (Figure 4). The volume and weight of the collected blood, the flow rate, and the time required for the donation process were consistently monitored. An audiovisual alarm system was used to promptly notify those involved of any issues related to blood flow or malfunction of the scale. In addition, an alarm was activated at 10 mL before the desired volume was reached and at the end of the donation process. After collecting the desired volume, an elastic cotton bandage was applied to the venipuncture site. The donor was monitored for the following 30 min for any complications. After confirming that hemostasis was achieved at the venipuncture site and that no hematoma had developed, the bandage was removed. After removing the bandage, the skin was grossly examined for contact dermatitis. A post-donation physical examination was then performed to detect any signs of hypovolemia or dehydration, after which the donor was offered treatment and water (Table 1).

**Table 1:** Demographics of the blood donation participants during the campaign.

Breed		Number	% of total
**Blood donation participant demographics**			
Labrador Retrievers		24	50.00
Golden Retrievers		5	10.42
Old English Sheepdogs		4	8.33
Samoyeds		3	6.25
Doberman Pinschers		3	6.25
German Shepherds		2	4.17
Standard Poodles		2	4.17
Sapsali		1	2.08
American Bully		1	2.08
Bernese Mountain Dog		1	2.08
Golden Doodle		1	2.08
Mixed breed		1	2.08
**Blood typing test for DEA 1**			
DEA 1 positive		31	67.39
DEA 1 negative		15	32.61
**Sex**			
Male	Intact male	3	11.54
	Castrated male	23	88.46
Female	Intact female	3	11.54
	Spayed female	19	86.36
**Age and body weight**			
Age (years)	Mean	3.58	
	SD	1.61	
Body weight (kg)	Mean	34.03	
	SD	5.95	

DEA, dog erythrocyte antigen; SD, standard deviation.

#### 2.4.3 Post-donation survey

During the post-donation monitoring period, the owners were asked to complete a questionnaire, the details of which are provided in Supplementary 1. The survey items were adapted from a previous study on human blood donation (13).

#### 2.4.4 Donor incentives

All canine donors underwent a comprehensive health assessment, which included a thorough physical examination and blood analysis, offered as a complementary pre-donation examination. In addition, incentives such as bandanas, vests, and donor ID cards were provided. In appreciation of their participation, a “hall of fame” section was set up on the campaign website, highlighting pictures of the donors during the campaign period.

### 2.5 Statistical methods

The collected data were subjected to descriptive statistical analyses that included the calculation of absolute and relative frequencies, means, and standard deviations (SDs) using Microsoft Office Excel 2013 software (Microsoft, United States). Statistical analyses were performed using SPSS version 28.0 (IBM Corp., Armonk, NY, United States). Data were presented as mean ± SD and were analyzed using a one-sample *t*-test. Statistical significance was considered at *p* < 0.05 in terms of indicating a difference between average satisfaction and overall response. A higher *t*-value indicates greater variance between the average scores of the survey group and the expected mean score.

## 3 Results

### 3.1 Promotion outcome and donor population analysis

Diverse communication channels were employed to promote the campaign, resulting in the recruitment of a total of 750 applicants before the targeted two-week recruitment period had elapsed. In addition, as a further promotional activity, emoticons that resembled donor dogs were created, which could augment the visibility of the campaign. These emoticons were then distributed to 20,000 supporters and could be used in the most popular messenger application in South Korea. On the campaign webpage, a total of 25,542 supporter comments were posted during the campaign. Moreover, there was a nearly 100-fold increase in the average volume of traffic to the Hyundai Motor Company website throughout the application period.

A total of 48 applicants were randomly selected. The blood donor dogs comprised the following breeds: 24 Labrador Retrievers (50%), 5 Golden Retrievers (10.42%), 4 Old English Sheepdogs (8.33%), 3 Samoyeds (6.25%), 3 Doberman Pinschers (6.25%), 2 German Shepherds (4.16%), 2 Standard Poodles (4.16%), 1 Sapsali (2.08%), 1 American Bully (2.08%), 1 Bernese Mountain Dog (2.08%), 1 Golden Doodle (2.08%), and 1 mixed breed (2.08%). The mean age (±SD) and mean body weight in kg (±SD) were 3.58 years (±1.61) and 34.03 kg (±5.95), respectively. Of the 26 males, 23 were neutered (88.46%) and 3 were intact (11.53%) and of the 22 females, 19 were spayed (86.36%) and 3 were intact (13.64%). Two dogs were unavailable for the blood test: one because of aggression and the other because of absence on the day. This left 46 dogs available for blood typing of DEA 1, among which 31 were DEA 1-positive (67.4%) and 15 were DEA 1-negative (32.6%).

Of the 48 participating dogs, 10 failed to proceed to blood collection for donation, which reduced the total number of blood donor dogs to 38. Among those that did not proceed to blood collection, two were excluded because of behavioral issues; five were ineligible because of positive results on point-of-care testing for vector-borne diseases; two were discontinued because of the formation of clots within the tubing system, resulting in a collection rate of less than 5 mL/min; and one was absent on the scheduled campaign date.

Of the 48 dogs participating in the study, 46 dogs were able to obtain blood sample for the Tick/Vector Comprehensive RealPCR^™^ Panel analysis. Two dogs tested positive on PCR analysis: one for *Bartonella* spp. and the other for *Mycoplasma* spp. Following the PCR results, blood products from these dogs were discarded. Notably, neither of these dogs produced positive results on the on-site SNAP^®^ 4Dx^®^ Plus tests.

### 3.2 In-procedure time and complications

On average, the pre-donation procedure took 30 min, blood collection took less than 15 min, and the post-donation procedure took 30 min. Considering collateral circumstances such as donor identification, post-donation surveys, and gift-giving, the participants stayed at the campaign location for approximately 90 min, mostly outdoors.

There were no donation-related complications, except for one dog that developed contact dermatitis induced by clipper irritation. All dogs were clinically healthy after blood donation and exhibited no clinical signs necessitating symptomatic treatment.

### 3.3 Post-donation survey

Out of a total of 48 on-site participants (the dog owners), 46 took part in the post-donation survey, with 2 individuals excluded. One person did not participate in the survey due to absence on the campaign day, while the other left before participating in the survey due to personal circumstances. According to a post-donation survey conducted using a 6-point scale (Supplementary 1), 93.5% of the participating owners expressed satisfaction (either fully or partly agreeing with positive statements) regarding the reduced travel time with their dogs. In addition, 95.7% answered that they hoped to participate again and 95.7% said they would recommend blood donation to other dog owners. Some criticism was made regarding the long waiting times, as 8.7% found that the total time they spent in relation to the campaign was too long. A total of 10.9% answered that the campaign should be better equipped to cater to the owners waiting for dogs (Table 2). A one-sample *t*-test was conducted to assess whether the average satisfaction rating significantly differed from the average score for each survey question. In this study, the average scores of those who provided positive responses did not exhibit a statistically significant difference when compared with the average scores of all respondents. This indicates that most respondents positively evaluated the campaign. Specifically, for questions that addressed negative assessments of the campaign (D, F, G, H, I, and J), the *p*-values were below 0.05, indicating that the respondents did not provide a negative response on average.

**Table 2:** Post-donation questionnaire contents and results regarding satisfaction with services and facilities of the mobile blood drive.

	Positive responses		Negative responses		Sample *T*-test results	
	Number	% of total	Number	% of total	*T*	*p*
A. Filling out the forms and screening questionnaires before donation really bothers me	0	0.00	46	100.00	0.065	0.474
B. I think the atmosphere at the blood drive is pleasant	43	93.50	0	0.00	0.971	0.169
C. The bloodmobile staff are extremely competent	42	91.30	0	0.00	0.226	0.411
D. Facilities at the bloodmobile should be better equipped to cater to the owners waiting for their dogs	5	10.90	2	4.30	5.471	0.003
E. I find it convenient to participate as the campaign location is nearby and my travel time is short	43	93.50	0	0.00	1.463	0.075
F. I spent too much time waiting for my dog’s blood donation	4	8.70	2	4.30	3.984	0.014
G. I think the compensation my dog/I receive for donating should be better	11	23.90	29	63.00	−9.139	<0.001
H. Coming to the blood drive takes considerable effort	0	0.00	38	82.60	−3.335	<0.001
I. I think that the notion of donating my dog’s blood is unpleasant	0	0.00	38	82.60	−3.303	0.001
J. The total time I had to spend today was too long	12	26.10	16	34.80	11.006	<0.001
K. I appreciate getting a small token “gift” for having my dog donate blood	46	100.00	0	0.00	0.056	0.478
L. I would like to volunteer my dog again	44	95.70	2	2.20	1.514	0.137
M. I would like to recommend blood donation to other dog owners	42	95.70	0	0.00	1.212	0.116

*p* < 0.05 indicates statistical significance.

In the short-answer section for additional comments, the most frequent positive responses were related to the rewarding feeling of helping patients in need of blood, improved accessibility of the blood bank, and encouragement for the use of ethical blood products. Two of the most notable drawbacks were the long outdoor waiting time and the narrow space inside the vehicle.

## 4 Discussion

Consistent with previous studies (4–6), we observed heightened awareness and recognition among pet owners regarding the importance of canine blood donation and the need for blood products in the veterinary field. Such increased recognition is anticipated to promote canine blood donor participation, thereby increasing potential blood product capacity in the future.

In a 2019 investigation of 158 pet owners’ awareness of small animal blood donation and blood banks, 70% were unaware of small animal blood donation and 75% were unaware of the existence of pet blood banks (6). Notably, 89% of the respondents expressed their willingness to participate after learning about pet blood donation. In this study, multiple modes of communication, such as television commercials, websites, and social media, were used for campaign promotion, resulting in the recruitment of 750 applicants in less than 2 weeks. This number is noteworthy, especially considering the distinct circumstances of South Korea, where the population of large dogs suitable for canine blood donation is considerably smaller than that of small dogs. It is estimated that large dogs constitute less than 10% of the overall companion dog population in the country (14).

This campaign was also of great significance because it raised public awareness of the need for blood transfusions in veterinary medicine. The vigorous promotion of the campaign proved exceptionally effective in boosting blood donation participation by informing pet owners about the existence of small animal blood donations and the significance of their participation. Consequently, it can be reasonably concluded that the prevailing scarcity of small animal blood donation is due to pet owners’ general lack of awareness rather than any unwillingness or reluctance to engage in blood donation (7). With the diligent and enthusiastic promotion of pet blood donations, more substantial blood donation participation and blood product availability can be anticipated.

The procedures used for the mobile blood drive in the current study were similar to those used in in-hospital blood donation programs. In general, there were no remarkable abnormalities in the results of the physical examination or bloodwork compared with in-hospital blood donation programs; however, 4 dogs produced false-positive results (8.33%) for *Anaplasma* spp. on the SNAP 4DX Plus test. A possible explanation is that human error occurred in unfamiliar settings. One dog developed contact dermatitis due to clipper irritation. The prevalence of DEA 1-positive and DEA 1-negative in the current study was consistent with that reported in previous studies (15–17). According to Carli et al., the ratio of DEA 1-positive to DEA 1-negative is approximately 6:4 (16). A recent large-scale study of the prevalence of DEA 1 in California revealed similar proportions of DEA 1 in that population (17).

Compared to regular in-hospital blood donation programs, this campaign took 2–3 times longer for the blood collection process. This extended collection time can be attributed to the unfamiliar environmental conditions that differ from the hospital setting and the limited space within the vehicle, which restricted the donor’s position to lateral recumbency. While blood collection is generally completed in as little as 6 min for a 320 mL donation in the hospital, during this campaign, it took an average of approximately 12 min. This limitation suggests the need for larger vehicles that provide greater inner space and more options for donor positions.

During the blood donation process, 10 out of 48 donor dogs failed to proceed to the blood collection stage. Among these 10 dogs, 2 had to discontinue donation due to clot formation within the blood collection tube, resulting in a collection rate of less than 5 mL/min. In humans, the four primary triggers for coagulation and clot formation in blood bags during donation are: a significant vascular endothelial injury caused by untidy or traumatic venipuncture; slow blood flow due to poor vein selection; overfilling or inadequate mixing of blood within the bag; and contamination with bacteria from inadequate skin cleansing (18). In this study, the blood clot formation probably occurred due to slow blood flow resulting from poor vein selection, as both donors exhibited a very slow collection rate (<15 mL/min) from the outset, despite the venipuncture being performed by skilled medical staff without any trauma.

In the present study, the two primary factors that significantly contributed to the highest satisfaction among the participating pet owners were emotional fulfillment derived from their dogs’ blood donation and the convenience of the mobile blood drive. The owners expressed both satisfaction following the blood donation process and a strong willingness to participate again in the future. Consistent with previous relevant research, it is evident that a sense of satisfaction derived from blood donation remains the foremost motivator for engagement, even among hesitant owners (19, 20). Furthermore, the predominant motivation, characterized by altruism and empathy among owners, aligns with that commonly observed in human blood donation (20, 21).

Blood donation is a worthy achievement, especially in assisting other animals in need of transfusion. This sense of achievement, which surpasses the appeal of material gifts or health checkup benefits, highlights the ethical, prosocial, and altruistic aspects of blood donation. Collectively, these findings suggest that fostering a consistently positive response before and after donating blood, raising awareness, and gaining public interest in veterinary blood donation will encourage more pet owners to participate as well as expand and secure the pool of potential donors.

In the post-program survey, participants’ satisfaction levels were notably high, especially considering that this was a first-time campaign. This success is expected to play a pivotal role in establishing blood donation practices among companion dogs. As interest in companion dog blood donation increases, a gradual increase in the number of participants is expected.

Another notable factor that contributed to participating pet owners’ satisfaction was the convenience offered by the mobile blood drive. As bloodmobiles can travel closer to the donor’s residence, the travel time and cost for the participant are significantly reduced. In addition, a short travel time alleviates the stress and anxiety associated with car rides for donor dogs. Bloodmobiles are beneficial and efficient, particularly for candidates unable to travel long distances, lacking adequate transportation, or experiencing severe transportation-related stress. The post-program survey revealed that increased accessibility owing to shorter travel times and distances was a point of satisfaction for most participants. Convenience is equally crucial in canine and human blood donation, as studies report that inconvenience is a major barrier to donating and contributes to donor loss (22, 23).

According to the post-program survey results, the two primary concerns were the limitations of the facilities and equipment, and the prolonged waiting time for the owners (Table 2). The bloodmobile used in this campaign had limited space, making it challenging to accommodate many people, including the owners. Although a waiting area was provided, extended waiting periods led to boredom, anxiety, and physical fatigue. Therefore, to improve the canine mobile blood drive and address the constraints identified in this study, a larger vehicle is required to accommodate a dedicated waiting area for owners and provide an expanded space for blood collection. Additionally, offering brief entertainment programs for owners, such as music and game sessions during extended waiting periods, can reduce complaints and alleviate anxiety (24, 25).

While the majority of the outcomes of this mobile blood drive were satisfactory, this study revealed several limitations and the need for further improvement. For instance, although the Hyundai Motor Company manufactures and provides high-quality blood donation vehicles for humans, the participating veterinary staff experienced some complications while using the modified vehicle. The temperature inside the vehicle was high, and the available space was confined. Although a large amount of power was required for the machines in the laboratory, electric sources were limited because the vehicle moved to multiple locations during this campaign. Occasional machine shutdowns occurred owing to unstable electrical supply. A portable generator was not sufficient to support all the machines, leaving the campaign staff to search for a steadier source of power. For future campaigns, the availability of a direct power supply should be prioritized when selecting campaign locations.

We assessed a canine bloodmobile that used a specially modified vehicle while conducting a national canine blood donation campaign on a trial basis. To the best of our knowledge, the bloodmobile described here is the first for companion dogs in Asia, and the second in the world after the University of Pennsylvania’s School of Veterinary Medicine (26). The results revealed that blood donation involving a bloodmobile could improve accessibility in terms of blood donation, increase the efficacy of blood donation programs by reducing travel distance and maximizing convenience for both owners and donors, and encourage further participation of canine blood donors owing to the effect of good publicity. Furthermore, actively promoting blood donation programs appears promising in terms of establishing a blood donation culture, as the public is still unfamiliar with the concept of canine blood donation.

## Data availability statement

The original contributions presented in the study are included in the article/Supplementary material, further inquiries can be directed to the corresponding author.

## Ethics statement

Ethical approval was not required for the studies involving animals in accordance with the local legislation and institutional requirements. The blood donation process outlined in this manuscript involved soliciting participation from guardians who expressed willingness to donate their dogs’ blood, obtaining consent from them, and proceeding accordingly. Furthermore, participating guardians received thorough explanations from veterinarians and ultimately provided their consent to participate by signing the consent forms. Therefore, no separate ethical approval was deemed necessary. Written informed consent was obtained from the owners for the participation of their animals in this study.

## Author contributions

H-JC: Conceptualization, Data curation, Investigation, Methodology, Software, Writing – original draft, Writing – review & editing. H-JH: Conceptualization, Methodology, Supervision, Writing – review & editing.
