# Supplementary material for: Launch of the first canine mobile blood donation center in Asia: development, outcomes, and influence of an animal bloodmobile
Source: Front Vet Sci. 2024 Jul 12;11:1402459. doi: 10.3389/fvets.2024.1402459 (PMC11272643; doi:10.3389/fvets.2024.1402459)
Supplement: Supplementary file 1 [file Table_1.docx]

Supplementary Material

**Supplementary 1.** The questionnaire was distributed to the owners during the post-donation monitoring period

| **Your views on the services and facilities at the “I'M DOgNOR” mobile blood drive** | | | | | | |
| --- | --- | --- | --- | --- | --- | --- |
| Below you will find a list of statements about the services and facilities at the blood bank. Please indicate the extent to which you agree or disagree with the statements below. If you don’t know which box to mark, you may use the option “Don’t know.” | | | | | | |
|  | **Fully disagree** | **Partly disagree** | **Neither agree  nor disagree** | **Partly agree** | **Fully agree** | **Don't know** |
|  | **1** | **2** | **3** | **4** | **5** | **6** |
| **A. It really bothers me to fill out the forms and screening questionnaires before donation.** |  |  |  |  |  |  |
| **B. I think the atmosphere at the blood drive is pleasant.** |  |  |  |  |  |  |
| **C. The bloodmobile staff are extremely competent.** |  |  |  |  |  |  |
| **D. Facilities at the campaign should be better equipped to cater to the owners waiting for their dogs.** |  |  |  |  |  |  |
| **E. I find it convenient to participate as the campaign location is nearby and my travel time is short.** |  |  |  |  |  |  |
| **F. I spent too much time waiting for my dog's blood donation.** |  |  |  |  |  |  |
| **G. I think the compensation my dog/I received for donating should be better.** |  |  |  |  |  |  |
| **H. Coming to the blood drive takes a lot of effort.** |  |  |  |  |  |  |
| **I. I think that the notion of donating my dog's blood is unpleasant.** |  |  |  |  |  |  |
| **J. The total time I had to spend today was too long.** |  |  |  |  |  |  |
| **K. I appreciate getting a small token "gift" for having my dog donate blood.** |  |  |  |  |  |  |
| **L. I would like to volunteer my dog again.** |  |  |  |  |  |  |
| **M. I would like to recommend blood donation to other dog owners.** |  |  |  |  |  |  |
| **Here you may write your views on any blood donation program services and bloodmobile facilities that are not mentioned above.** | | | | | | |
|  | | | | | | |
